# Time-Resolved Laser-Induced Breakdown Spectroscopy for Accurate Qualitative and Quantitative Analysis of Brown Rice Flour Adulteration

**DOI:** 10.3390/foods11213398

**Published:** 2022-10-27

**Authors:** Honghua Ma, Shengqun Shi, Deng Zhang, Nan Deng, Zhenlin Hu, Jianguo Liu, Lianbo Guo

**Affiliations:** 1 Wuhan National Laboratory for Optoelectronics (WNLO), Huazhong University of Science and Technology, Wuhan 430074, China; 2 School of Physics and Electronic Information Engineering, Hubei Engineering University, Xiaogan 432000, China

**Keywords:** laser-induced breakdown spectroscopy, brown rice flour adulteration, time-resolved spectra, machine learning, deep learning

## Abstract

To solve the adulteration problem of brown rice flour in the commodity market, a novel, accurate, and stable detection method based on time-resolved laser-induced breakdown spectroscopy (TR-LIBS) is proposed. Qualitative and quantitative analysis was used to detect five adulterants and seven different adulterant ratios in brown rice flour. Being able to excavate more information from plasma by obtaining time-resolved spectra, TR-LIBS has a stronger performance, which has been further verified by experiments. For the qualitative analysis of adulterants, the traditional machine learning models based on TR-LIBS, linear discriminant analysis (LDA), naïve Bayes (NB) and support vector machine (SVM) have significantly better classification accuracy than those based on traditional LIBS, increasing by 3–11%. The deep learning classification model based on TR-LIBS also achieved the same results, with an accuracy increase of more than 8%. For the quantitative analysis of the adulteration ratio, compared with traditional LIBS, the quantitative model based on TR-LIBS reduces the limit of detection (LOD) of five adulterants from about 8–51% to 4–19%, which effectively improves the quantitative detection performance. Moreover, t-SNE visualization proved that there were more obvious boundaries between different types of samples based on TR-LIBS. These results demonstrate the great prospect of TR-LIBS in the identification of brown rice flour adulteration.

## 1. Introduction

Food adulteration refers to artificially and purposefully adding diverting ingredients to food, such as for preservation, color enhancement, improvement of appearance, texture and masking, to achieve the purpose of improving economic benefits [1]. Rice, the staple food for more than 3.5 billion people, can be processed to produce brown and white rice [2,3]. Compared with white rice, brown rice is rich in starch, protein, fat, vitamins, minerals, and functional health care ingredients, and also contains dietary fiber, oryzanol, glutathione, γ-aminobutyric acid, rice bran polysaccharide. It can provide more comprehensive nutrition, which is very suitable for people with obesity, gastrointestinal dysfunction, anemia, constipation and diabetes [4,5]. Therefore, the sales of brown rice are on a continuous rise worldwide [6]. However, many unscrupulous suppliers adulterate brown rice in pursuit of more profits, which may lead to decreased nutritional value, human health problems, and even death in serious cases [7]. Therefore, rice flour adulteration detection is an important way to ensure food safety.

Plenty of studies have provided various approaches for adulteration identification of rice products, including a DNA-based method combined with High Resolution Melting (HRM) [8], inductively coupled plasma mass spectrometry (ICP-MS) and isotope ratio mass spectrometry (IRMS) [9,10,11,12]. Similarly, several studies have shown that gas chromatography-mass spectrometry (GC-MS) and liquid chromatography-mass spectrometry (LC-MS) are useful as certification methods for organic rice to detect adulterated components in high-quality rice [13,14,15,16,17]. However, they require sample digestion, which means a long detection period, high requirements for the operation environment, and environmental pollution caused by the use of chemical reagents [18]. In addition, recent studies have shown that spectrum-based detection methods are also applicable to food safety monitoring. For instance, Attaviroj et al. proposed the application of Fourier-transform near-infrared spectroscopy (FT-NIR) to identify moist brown rice varieties [19]. Li et al. proposed terahertz (THz) spectroscopy combined with support vector machine (SVM) to identify adulterated rice [20]. Hyperspectral imaging technology (HSI) can also identify both spatial and chemical information of wheat flour products [21]. Nevertheless, the above spectral detection methods also have some disadvantages. The liquid composition in the sample affects the analytical performance of the NIR and THz spectra. In addition, NIR requires a large number of representative known chemical values for modeling. HSI data is large and redundant, and processing is very complex. Therefore, a simple, rapid, and in situ method for distinguishing brown rice from adulterated samples is urgently needed to ensure food safety in the grain market.

Laser-induced breakdown spectroscopy (LIBS) has become a well-established and powerful optical emission spectroscopy analysis technology after more than 60 years of development. It has been praised as the “future super star” in analytical chemistry with the advantages of multi-element analysis, fast response, remote detection and no or simple preparation [22]. In recent years, LIBS has been gradually applied to the field of food detection, including monitoring calcium content in comminuted poultry meat [23], adulteration detection of milk powder [24,25], and classification of red wine based on its protected designation of origin (PDO) [26]. However, few LIBS works were reported on the quality detection of brown rice products. Ribeiro et al. analyzed the composition of rice varieties by LIBS combined with Fourier-transform infrared spectroscopy (FTIR) [27]. Yang et al. proved that LIBS combined with chemometrics can distinguish the geographical origin of different rice [28]. Pérez-Rodríguez et al. carried out significant research on brown rice detection. They used spark discharge-LIBS (SD-LIBS) combined with the k-nearest neighbor (KNN) to distinguish the PDO certification of brown rice [7]. These representative works on LIBS detection of rice demonstrate the potential for rapid identification of adulterated products without chemical digestion. However, the LIBS spectra are unstable due to the influence of sample morphology, laser energy and other factors, and this fluctuation leads to a decrease in the accuracy of qualitative analysis. In addition, detection methods similar to SD-LIBS will lead to increased operational complexity. The above factors seriously restrict the further development of LIBS in the field of rice flour adulteration identification. Therefore, the detection of different adulterants in rice products by LIBS needs to be further improved.

In this work, a new simplified time-resolved LIBS (TR-LIBS) was developed for accurate qualitative and quantitative analysis of brown rice flour adulteration based on [25]. TR-LIBS technology does not require sample digestion. Compared to NIR and HSI technologies, it has a lower data dimension and does not require a large number of representative chemical values for modeling. Moreover, TR-LIBS can effectively mine the key information such as intensity and its evolution over delay time by obtaining time-resolved spectra, so as to enhance the accuracy and robustness of the analytical models. To verify the effectiveness of TR-LIBS, we compared it with traditional LIBS from the perspective of qualitative and quantitative analysis of adulteration. The results proved that TR-LIBS is an accurate, reliable, and stable analytical method.

## 2. Materials and Method

### 2.1. Materials and Sample Preparation

In the commodity market, products with low nutritional value are often mixed with products with high nutritional value. As a kind of food with high nutritional value, brown rice flour is always mixed with some low-priced food to obtain additional economic benefits, such as sorghum flour, talc powder and corn flour. Samples used in this work include sorghum flour (SF), talc powder (TP), corn flour (CF), buckwheat flour (BF), gypsum powder (GP), and brown rice flour (BRF), all of which were purchased from the market in China. ICP-MS was used as a reference method to determine the elemental composition of these six kinds of samples, and the results in Table 1 show that TP and GP are significantly different from SF, CF, BF, and BRF, while SF, CF, BF, and BRF are similar. Appropriate amounts of SF, TP, CF, BF and GP were mixed into BRF to achieve the target concentration: 1%, 3%, 5%, 8%, 10%, 15%, 20% and 25% (*w*/*w*). Five grams of mixed powder was pelleted by electric tablets press applying a pressure of 30 tons for 1 min. The thickness and diameter of the pellets were about 3 mm and 40 mm, respectively. To eliminate individual differences in samples, two repeated samples were made for each concentration gradient. A total of 82 pressed pellets were prepared for LIBS measurement without further treatment.

### 2.2. LIBS Setup and Measurement

**Figure 1:**
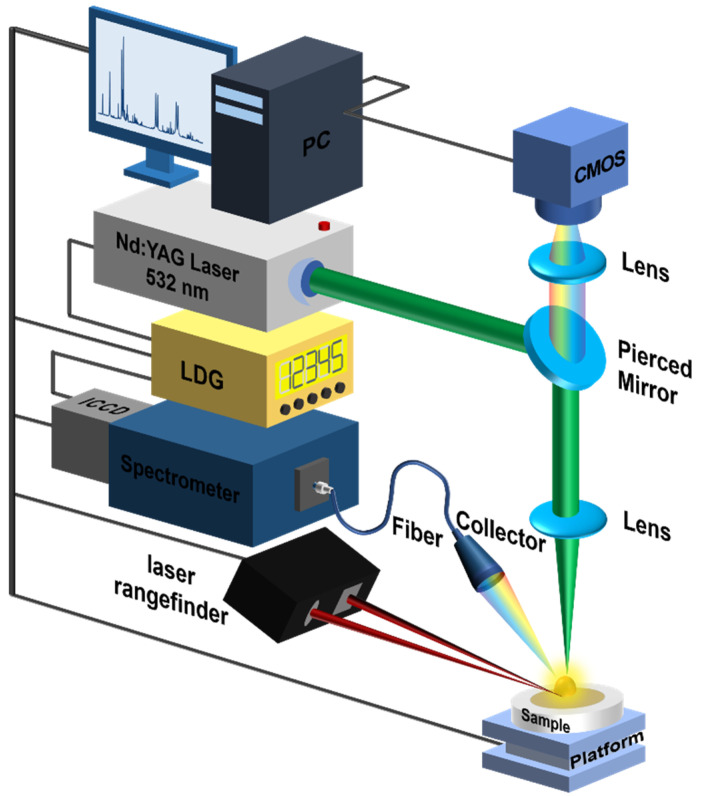
LIBS rice flour detection system device schematic.

The traditional LIBS device was used in this work. A laser pulse from a Q-switched Nd: YAG laser (Nimma-400, wavelength: 532 nm; pulse duration: 8 ns; flattened Gaussian beam, Beamtech Optronics Co., Ltd., Beijing, China) is focused on the sample, which is placed on a three-dimensional electric displacement platform (DZY110TA-3Z, Beijing Jiangyun Juli Technology Co., Ltd., Beijing, China), after passing through a quartz lens to generate plasma. The movement of the x-y-z platform is controlled by the laser rangefinder ( CDX-85A, Aotaisi Industrial Automation Control Equipment Co., Ltd., Guangdong, China). The plasma emission is detected by the spectrometer (Mechelle 5000, resolution: λ⁄∆λ = 5000; spectral range: 200–950 nm; Andor Technology Ltd., Belfast, United Kingdom) via a light collector and UV-enhanced fiber optic with a 50 μm core. The whole system is controlled by using a self-designed LIBS digital delay generator (LDG 3.0, Wuhan N&D Laser Engineering Co., Ltd., Wuhan, China). LIBS detection system device schematic is shown in Figure 1.

**Figure 2:**
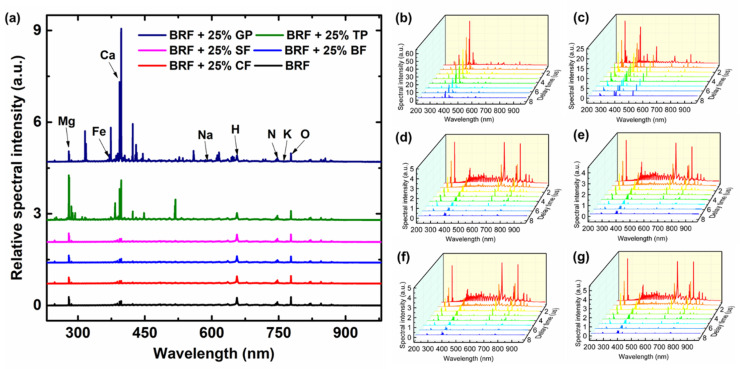
(**a**) LIBS spectra and (**b**–**g**) time-resolved spectra of BRF with 25% GP, BRF with 25% TP, BRF with 25% SF, BRF with 25% BF, BRF with 25% CF and BRF, where the lines with different colors in (**b**–**g**) represent the spectra with different time delay.

In this work, the optimal experimental parameters were as follows, the laser energy was set as 40 mJ and the repetition frequency was 1 Hz. The gate width and exposure time of the spectrometer were fixed to 2 μs and 0.101 s, respectively. The gate delay was set from 1 μs to 4.5 μs, with 0.5 μs as a step to obtain the time-resolved spectra. All samples were measured in the atmosphere. The LIBS spectra (gate delay: 1 μs; gate width: 2 μs) of 25% adulteration were shown in Figure 2a. In addition, time-resolved spectra of BRF and its adulterated samples are also shown in Figure 2. For each pellet, 15 groups of time-resolved spectra (120 LIBS spectra without accumulation and average) were obtained. Different adulterations (1%, 3%, 5%, 8%, 10%, 15% and 25%) of the same mixture were regarded as the same class, and all samples were divided into six classes including BRF + SF (class 1), BRF + TP (class 2), BRF + CF (class 3), BRF + BF (class 4), BRF + GP (class 5) and BRF (class 6). A total of 1230 groups of time-resolved spectra were used for subsequent qualitative and quantitative analysis.

### 2.3. Data Analysis

Traditional LIBS analysis only focuses on the spectra radiated by plasma at a certain gate delay and gate width, which results in only the element intensity information under this state being obtained, while a large amount of useful information of plasma is lost. To solve this problem, a novel time-resolved laser-induced breakdown spectroscopy (TR-LIBS) is proposed in this work. After obtaining LIBS spectra under multiple delay times, this method extracted features and spliced them, and finally inputted them into the analysis models. Next, the process of TR-LIBS is described in detail. As can be seen from Figure 2, the spectra of these samples are sparse, and most wavelengths are redundant. Therefore, spectral feature selection or dimensionality reduction is needed. In this work, 64 lines were selected, mainly including lines of nitrogen (N), oxygen (O), hydrogen (H), carbon (C), calcium (Ca), sodium (Na), potassium (K), magnesium (Mg) and other elements according to ICP-MS results and National Institute of Standards and Technology (NIST) atomic spectral database. After feature selection, eight time-resolved spectra were spliced to form a 512-length one-dimensional sequence, which was used as the input of the analysis models.

To verify the effectiveness of TR-LIBS in improving qualitative model analysis performance, LIBS and TR-LIBS were compared based on the traditional machine learning model and deep learning model. For traditional machine learning models, three commonly used models, linear discriminant analysis (LDA), Naive Bayes (NB), and support vector machines (SVM) were selected. LDA is a supervised machine learning algorithm. It can find the optimal representation of data in low dimensions by maximizing the inter-class divergence matrix and minimizing the intra-class divergence matrix, and can effectively extract classification features. The NB algorithm is a method based on Bayes theorem and independence hypothesis of characteristic condition, which needs fewer parameters and is not easy to disturb outliers. The SVM algorithm is based on statistical learning theory and can maximize the interval between data while minimizing the empirical error. It is also a classical supervised classification algorithm. It uses a kernel function to transform the linear inseparable problem of low dimensional space into the linear separable problem of high dimensional space and then realizes the accurate classification of data. These methods are also the most common and effective analytical methods in spectral analysis [29,30]. For the deep learning model, a one-dimensional convolutional neural network (1D-CNN) was constructed. The core idea of CNN is the sparse connection, weight sharing and pooling sampling. Through convolution operation, the original signal features can be enhanced and the noise can be reduced. The pooling operation uses the principle of local correlation of the image to downsample the image, which can reduce the amount of data processing while retaining useful information. CNN also achieves excellent performance in LIBS analysis [31].

In addition, to verify the effectiveness of TR-LIBS in improving quantitative analysis performance, a partial least squares regression (PLSR) model [32] was established on seven different adulteration ratios of five adulterants. PLSR is a linear multivariate data analysis method based on factor analysis, which extracts components from the independent variables that have both higher generalization of information on the independent variable system and better interpretation of the dependent variable and determines the number of principal factors, and then builds a regression model of the principal factors and the dependent variable.

### 2.4. Evaluation Indexes

The qualitative and quantitative performance of the traditional LIBS and our proposed TR-LIBS method were compared in detail through several evaluation indexes. Qualitative analysis is mainly evaluated by accuracy and confusion matrix. Different from qualitative analysis, the performance evaluation indexes for quantitative analysis mainly include the determination coefficient (*R*^2^), the root-mean-square error (RMSE), and the average relative error (ARE) [33]. This work also calculated the limit of detection (LOD) of five adulterants under PLSR [34]. Their expressions are as follows:

(1) $$Accuracy=\frac{100}{n}\overset{n}{\underset{i=1}{\sum }}\delta_{i}$$

(2) $$R^{2}=1-\frac{\sum_{i=1}^{n}{\left({\hat{y}}_{i}-y_{i}\right)}^{2}}{\sum_{i=1}^{n}{\left({\hat{y}}_{i}-\bar{y}\right)}^{2}}$$

(3) $$\mathrm{RMSE}=\sqrt{\frac{\sum_{i=1}^{n}{\left({\hat{y}}_{i}-y_{i}\right)}^{2}}{n}}$$

(4) $$\mathrm{ARE}=\frac{100}{n}\overset{n}{\underset{i=1}{\sum }}\frac{\left|{\hat{y}}_{i}-y_{i}\right|}{y_{i}}$$

(5) $$\mathrm{LOD}=3.3s_{pu}^{-1}{\left[\left(1+h_{0min}+\frac{1}{n}\right)var_{pu}\right]}^{\frac{1}{2}}$$

where *n* is the number of samples, $\delta_{i}$ is either 1 when a spectrum is classified correctly or 0 otherwise, $y_{i}$ is the certified concentration of the *i*_th_ sample, $\bar{y}$ is the average value of $y_{i}$ over *n* sample, ${\hat{y}}_{i}$ is the predicted concentration of the *i*_th_ sample, *s_pu_* is the slope of the Pseudounivariate line, *h*_0*min*_ is the minimum projected leverage for a blank sample, *var_pu_* is the variance of the regression residuals.

## 3. Results of Qualitative and Quantitative Analysis

In this work, the validity of TR-LIBS was verified by spectral analysis of BRF and its adulterated samples. To solve data problems such as imbalance, the Synthetic Minority Over-sampling Technique (SMOTE) [35] approach was utilized. Before modeling, the spectra were randomly divided into the training set and test set according to the ratio of 7:3. The training set was used to train the models and optimize the hyperparameters of the models. The test set was used to validate the optimized models. To eliminate the influence of the randomness of sample division, 20 random segmentation operations were performed, and the average accuracy of models was obtained by averaging the 20 test results. The qualitative and quantitative analysis results of LIBS and TR-LIBS in brown rice flour and adulterants were introduced below.

### 3.1. Qualitative Analysis of Adulterants

#### 3.1.1. The Results of Traditional Machine Learning Models

**Figure 3:**
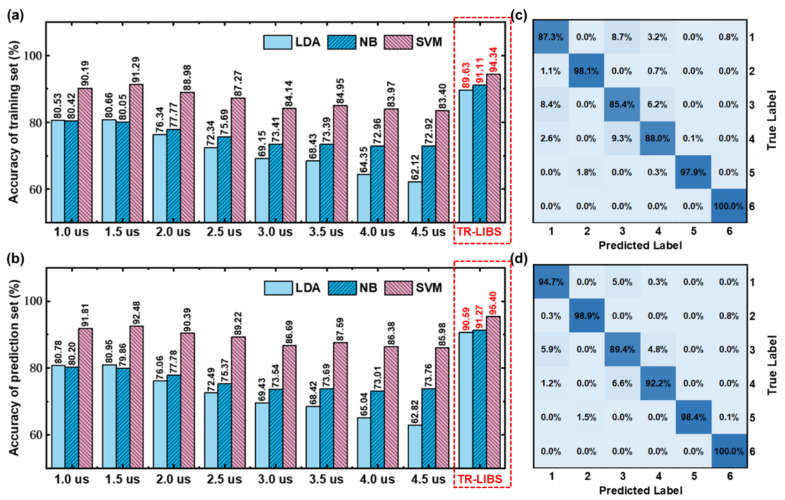
(**a**) Training set and (**b**) test set results of LDA, NB and SVM models based on LIBS spectra and time-resolved spectra, and the test set confusion matrix of SVM models based on (**c**) LIBS spectra under 1.5 μs and (**d**) time-resolved spectra.

To make the results universal, three traditional machine learning classification models, LDA, NB, and SVM, are used for analysis. First, these six types of samples are classified based on traditional LIBS. Traditional LIBS only uses a single spectrum of each sample for classification. In this work, LIBS spectra are obtained at eight delay times, such as 1 μs, 1.5 μs, 2 μs, 2.5 μs, 3 μs, 3.5 μs, 4 μs, and 4.5 μs. Therefore, after feature selection, eight classification models are established based on the spectra obtained under different delay times, and the results are shown in Figure 3a,b. It can be seen that the performance of classification models established under different delay times is obviously diverse. The training set accuracy of LDA classification models is established under different delay times from 62.12% to 80.66%, and the test set accuracy from 62.82% to 80.95%. The training set accuracy of NB classification models is 72.92–80.42%, and the test set accuracy is 73.01–80.20%. The training set accuracy of SVM classification models is 83.40–91.29%, and the test set accuracy is 85.98–92.48%. It can be seen from the results that the accuracy of the classification models increases first and then decreases with the increase in delay time. This is due to the continuum emission of the initial plasma being very strong, where the atomic and ion spectral lines are submerged. With the evolution of the plasma, the continuum emission intensity decreases, the intensity of atomic and ionic spectral lines increases, and the effective information of plasma can be fully mined. However, with further evolution, the plasma gradually annihilates, and the intensity of atomic and ionic spectral lines decreases or even disappears. The classification performance of SVM is significantly higher than LDA and NB. Among them, the SVM model established under the delay time of 1.5 μs has the optimal performance. The training set accuracy of this model is 91.29%, and the accuracy of the test set is 92.48%. The training set confusion matrix is shown in Figure 2c. The misclassification rate between class 1, 3, and 4 is very high. It can be seen from the ICP-MS results that the very similar type and concentration of elements among SF, CF, and BF are the reasons for this phenomenon.

Furthermore, TR-LIBS is utilized to classify these six kinds of samples. After feature selection, spectral features under different delay times are spliced and finally input into LDA, NB, and SVM classification models. The results of classification models based on TR-LIBS are shown in Figure 3a,b. The training set accuracy of the LDA classification model is 89.63%, and the test set accuracy is 90.59%. The training set accuracy of the NB classification model is 91.11%, and the test set accuracy is 91.27%. The training set accuracy of the SVM classification model is 94.34%, and the test set accuracy is 95.40%. The optimal classification model based on TR-LIBS is still SVM. The accuracy of classification models based on TR-LIBS is obviously better than those based on traditional LIBS, which is increased by about 3–11%. As can be seen from the confusion matrix of the test set in Figure 3d, the misclassifications among the six classes were significantly reduced, especially between classes 1, 3, and 4. These results preliminarily demonstrate the effectiveness of TR-LIBS in improving qualitative analysis performance.

#### 3.1.2. The Results of Deep Learning Models

**Figure 4:**
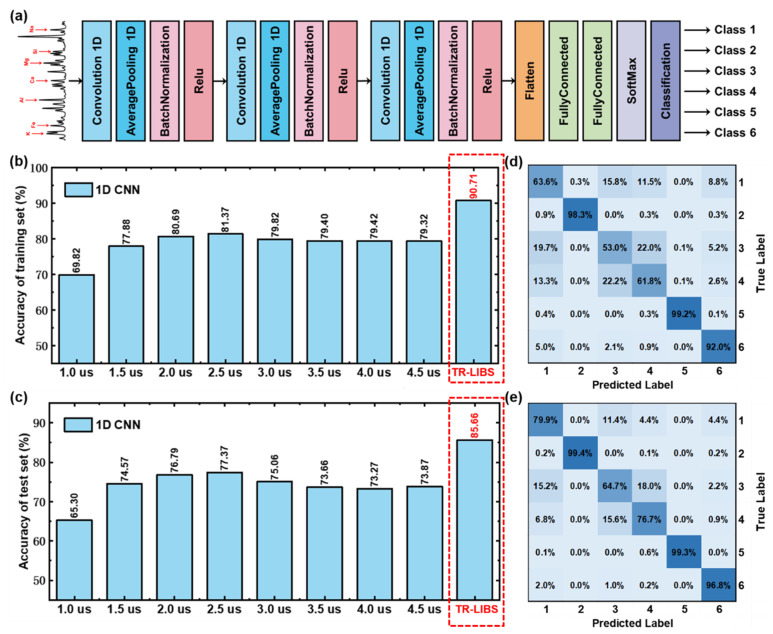
(**a**) Structural diagram of 1D CNN model, (**b**) training set and (**c**) test set results of 1D CNN models based on LIBS spectra and time-resolved spectra, and the test set confusion matrix of 1D CNN models based on (**d**) LIBS spectra under 2.5 μs and (**e**) time-resolved spectra.

Furthermore, the convolutional neural network in the deep learning model is used to compare the performance of traditional LIBS and TR-LIBS. As shown in Figure 4a, an 18-layer one-dimensional convolutional neural network (1D CNN) is built after structure and parameter optimization in this work. Three convolution layers, three average pooling layers, and three Relu activation layers are used. The size of the convolution layer is 1 × 3, and the size of the average pooling layer is 1 × 5. The number of convolution kernel is set as 8, 8, and 8, respectively. To prevent over-fitting, the batch normalization layer is placed before the activation layer. A flatten layer and two full connection layers are used to map the feature space computed by the front layer to the sample marker space. The number of neurons in the full connection layer is set to 20 and 6, respectively. The activation function of the output layer is Softmax. The hyperparameters of the 1D CNN model are Epoch of 60, Batchsize of 128, and Learning Rate of 0.0005.

This constructed 1D CNN is used to identify these six types of samples based on traditional LIBS. Similarly, 1D CNN classification models are established based on the spectra obtained under different delay times, and the results are shown in Figure 4b,c. The accuracy of the training set ranges from 69.82% to 81.37%, and that of the test set ranges from 65.30% to 77.37%. The evolution of 1D CNN classification model accuracy with delay time is consistent with that of the traditional machine learning model. However, the delay time corresponding to the optimal model is different. The 1D CNN model at 2.5 μs has the best performance. The accuracy of the training set and the test set is 81.37%, and 77.37%, respectively. Compared with traditional machine learning models, 1D CNN model has poor performance, which may be caused by the small amount of data. Convolutional Neural Network is a deep model with numerous parameters, requiring more data for parameter optimization and learning. The test set confusion matrix is shown in Figure 4d, and the misclassification between classes 1, 3, and 4 is very obvious.

Furthermore, a 1D CNN classification model is trained based on TR-LIBS, and the results are shown in Figure 4b–e. The accuracy of the training set and test set of this 1D CNN classification model is 90.71% and 85.66%, respectively. It can be seen that compared with traditional LIBS, the performance of 1D CNN model is significantly improved, and the test set accuracy is increased by more than 8%. Finally, we compare the classification accuracy of the traditional LIBS and TR-LIBS combined with machine learning models and deep learning models on the training and prediction set, respectively, in Table 2. The above experimental results fully verify the effectiveness of TR-LIBS in improving qualitative analysis performance from two aspects of traditional machine learning models and deep learning models.

### 3.2. Quantitative Analysis of the Adulteration Ratio

Based on the above qualitative analysis of adulterants, seven adulterant ratios (1, 3, 5, 8, 10, 15, and 25%) of five adulterants are further quantitatively analyzed in this section. The adulteration is composed of different elements. Therefore, the quantitative analysis of the ratio is a multivariate regression process. In this work, the PLSR model is used to fit the ratio of adulteration.

Data preprocessing for quantitative analysis for LIBS and TR-LIBS is the same process as the qualitative analysis, but the supervision label changes from the adulteration category to the adulteration ratio. Based on the above evaluation metrics, the quantitative performance of traditional LIBS and TR-LIBS on the training set and prediction set of five adulterations is compared in detail in Table 3. For the LIBS method, this section selects the best results under eight different delays. The data show that the quantitative results of TP are the best. The LOD of the PLSR model based on LIBS is 7.930%, and the determination coefficient of prediction (*R*^2^_P_), root-mean-square error of prediction (RMSE_P_) and average relative error of prediction (ARE_P_) are 0.963, 1.563% and 27.578%, respectively. The corresponding results of the PLSR model based on TR-LIBS are 4.175%, 0.977, 1.312% and 24.509%, respectively. However, the quantitative results of CF are relatively poor, the LOD of the PLSR model based on LIBS is 51.148%, and the *R*^2^_P_, RMSE_P_ along with ARE_P_ are 0.308, 5.945% and 127.231%, respectively. The corresponding results of the PLSR model based on TR-LIBS are 18.680%, 0.701, 4.011% and 61.823%, respectively. The quantitative results of the PLSR model based on TR-LIBS and LIBS are consistent with the ICP-MS element detection results. The results of ICP-MS show that the content of Ca in TP and GP is higher, which is significantly different from that of BRF, while the element levels of SF, CF and BF are less different from those of BRF. In addition, the quantitative results also show that, compared with LIBS, the model based on TR-LIBS has improved all evaluation indexes on the training set and the prediction set, and the model performance is better.

**Figure 5:**
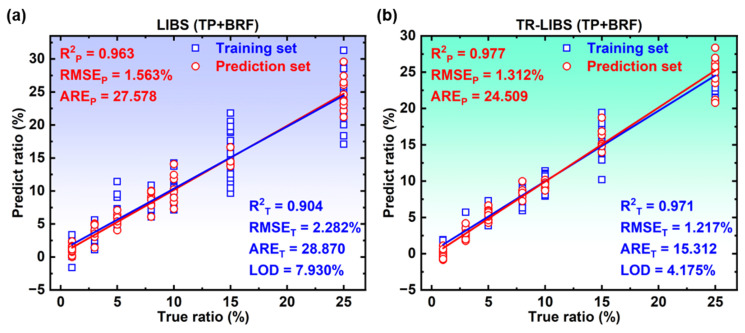
Linear fitting results of the PLSR model based on (**a**) LIBS and (**b**) TR-LIBS in the training set and prediction set of adulterant TP.

To further analyze the quantitative performance of TR-LIBS and LIBS, the quantitative performance of PLSR on the two methods is compared by linear fitting with the adulteration TP as the representative in this section. The results are shown in Figure 5. The above experimental results prove the effectiveness of TR-LIBS in quantitative analysis.

**Table 3:** Comparison of quantitative analysis results of five adulterants in BRF by LIBS and TR-LIBS. The subscript value is the corresponding optimal delay.

Adulteration Samples	Method	LOD (%)	Training Set			Prediction Set		
			*R* ^2^_T_	RMSE_T_ (%)	ARE_T_ (%)	*R* ^2^_P_	RMSE_P_ (%)	ARE_P_ (%)
sorghum flour (SF)	LIBS_(4.5)_	20.357	0.662	4.340	58.528	0.606	5.017	69.303
	TR-LIBS	**12.665**	0.832	3.137	56.326	0.782	3.507	70.675
talc powder (TP)	LIBS_(2.0)_	7.930	0.904	2.282	28.870	0.963	1.563	27.578
	TR-LIBS	**4.175**	0.971	1.217	15.312	0.977	1.312	24.509
corn flour (CF)	LIBS_(1.5)_	51.148	0.414	5.992	124.777	0.308	5.945	127.231
	TR-LIBS	**18.680**	0.721	4.095	88.590	0.701	4.011	61.823
buckwheat flour (BF)	LIBS_(1.5)_	19.138	0.722	4.103	74.164	0.536	4.945	87.205
	TR-LIBS	**11.184**	0.861	2.886	46.862	0.822	3.027	44.806
gypsum powder (GP)	LIBS_(1.5)_	8.419	0.885	2.556	37.408	0.895	2.547	32.365
	TR-LIBS	**4.492**	0.968	1.311	19.374	0.974	1.321	21.409

## 4. Discussion

**Figure 6:**
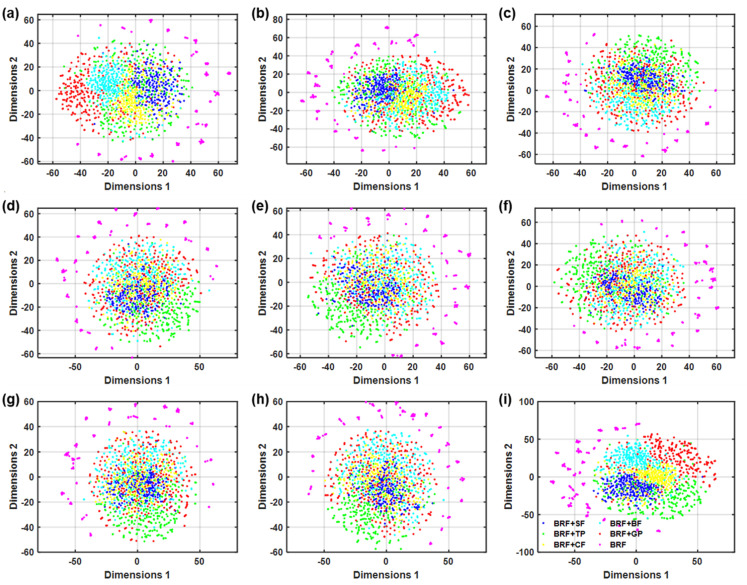
Results of t-SNE visualization based on spectra under (**a**) 1, (**b**) 1.5, (**c**) 2, (**d**) 2.5, (**e**) 3, (**f**) 3.5, (**g**) 4 and (**h**) 4.5 μs and (**i**) time-resolved spectra.

This section will discuss in detail the main reasons why TR-LIBS is better than traditional LIBS. Firstly, in addition to the spectral intensity information of elements, the evolution of spectral intensity is one of the important information in plasma. TR-LIBS can effectively mine this information by acquiring time-resolved spectra. Secondly, TR-LIBS can effectively reduce the influence of plasma fluctuations caused by external disturbances by acquiring multiple spectra, thus having stronger robustness. In summary, TR-LIBS improves qualitative and quantitative analysis performance by excavating more information from plasmas. To make the explanation more convincing, the t-distributed stochastic neighbor embedding (t-SNE) algorithm is utilized for the visual analysis of traditional LIBS and TR-LIBS data. The visualization results are shown in Figure 6. It can be seen that for the traditional LIBS, there is a serious overlap between the spectra of different samples and no obvious class boundaries. For TR-LIBS, the spectra of the same sample are more aggregated, and the spectra of different samples are more dispersed with obvious boundaries.

The LIBS spectral levels of the same element in different matrices are different. Therefore, the matrix effect has always been considered as one of the problems in the qualitative and quantitative analysis of LIBS [36]. However, in TR-LIBS, the matrix can be regarded as effective information as a potential feature for qualitative and quantitative analysis. Since the time-resolved spectra of the same element in different matrices have different trends. In addition, compared with the LIBS spectrum collected under the traditional single time series, the time-resolved LIBS collected under one time series has stronger resistance to fluctuation. These factors are of great significance to the qualitative and quantitative analysis of substances. The above principle analysis once again proves the effectiveness of TR-LIBS in improving the performance of the qualitative and quantitative analysis, which is of positive significance for promoting the development of LIBS in other fields such as environmental pollution detection.

According to the results of qualitative and quantitative analysis, this study also shows some limitations. The element level of the adulterated material is too close to that of the original blank sample, which will have a negative impact on the qualitative and quantitative performance of the model. This issue is also one of the directions that LIBS needs to further research in the field of food safety control in the future.

## 5. Conclusions

To realize the high precision identification of brown rice flour adulteration, this work proposes a novel method named TR-LIBS. TR-LIBS can excavate more effective information from plasma by obtaining time-resolved spectra, to improve the performance of the qualitative and quantitative analysis. This study fully verifies the effectiveness of the method from two aspects of adulterant classification and adulterant proportion quantification. For the qualitative classification of adulterants, the results of three traditional machine learning models (LDA, NB and SVM) are compared. The results show that the accuracy of the machine learning models based on TR-LIBS is significantly better than that of the machine learning models based on traditional LIBS, which is improved by about 3–11%. Moreover, for the qualitative classification of deep learning models, the test set accuracy of 1D CNN based on TR-LIBS is improved from 77.37% to 85.66% compared with traditional LIBS, an increase of more than 8%. For the quantitative analysis of the proportion of adulteration, the results of PLSR models based on traditional LIBS and TR-LIBS are compared. The results show that all the performance evaluation indexes of the model based on TR-LIBS are significantly better than those based on traditional LIBS. The LOD based on LIBS was about 8% to 51%, while the LOD of the five adulterants based on TR-LIBS was reduced from about 4% to 19%. This indicates that TR-LIBS significantly improves detection performance. Finally, visual analysis of spectra based on the t-SNE algorithm shows that there are obvious boundaries between different types of samples in TR-LIBS, while traditional LIBS does not. These results demonstrate that TR-LIBS is a reliable and stable high-precision qualitative and quantitative analysis method, which is of great significance for promoting the further application of LIBS in various fields.

## Figures and Tables

**Table 1:** The elemental concentration of six kinds of samples were measured by ICP-MS.

Samples	Elemental Concentration (mg/kg)							
	Ca	Cu	Fe	K	Mn	Na	P	Zn
SF	93.88	2.14	28.04	2332.01	12.68	14.61	2600.04	17.00
TP	16,387.98	1.08	3655.71	207.02	102.65	118.67	171.52	4.21
CF	34.15	0.59	8.95	1127.84	0.63	9.48	773.68	6.70
BF	131.17	3.14	21.14	2965.93	8.78	11.61	3138.66	16.78
GP	249,581.83	0.70	47.09	62.84	0.31	49.86	4.01	7.50
BRF	97.07	2.19	10.66	1376.19	35.31	14.51	1702.35	16.23

**Table 2:** Classification accuracy of traditional LIBS and TR-LIBS combined with machine learning models and deep learning models on the training and prediction set.

Method	Delay	Accuracy of the Training Set (%)				Accuracy of the Prediction Set (%)			
		LDA	NB	SVM	1D-CNN	LDA	NB	SVM	1D-CNN
Traditional LIBS	1 μs	80.53	80.42	90.19	69.82	80.78	80.20	91.81	65.30
	1.5 μs	80.66	80.05	91.29	77.88	80.95	79.86	92.48	74.57
	2 μs	76.34	77.77	88.98	80.69	76.06	77.78	90.39	76.79
	2.5 μs	72.34	75.69	87.27	81.37	72.49	75.37	89.22	77.37
	3 μs	69.15	73.41	84.14	79.82	69.43	73.54	86.69	75.06
	3.5 μs	68.43	73.39	84.95	79.40	68.42	73.69	87.59	73.66
	4 μs	64.35	72.96	83.97	79.42	65.04	73.01	86.38	73.27
	4.5 μs	62.12	72.92	83.40	79.32	62.82	73.76	85.98	73.87
TR-LIBS	—	**89.63**	**91.11**	**94.34**	**90.71**	**90.59**	**91.27**	**95.40**	**85.66**

## Data Availability

The datasets generated for this study are available on request to the corresponding author.
